# A fluoroxalate cathode material for potassium-ion batteries with ultra-long cyclability

**DOI:** 10.1038/s41467-020-15044-y

**Published:** 2020-03-06

**Authors:** Bifa Ji, Wenjiao Yao, Yongping Zheng, Pinit Kidkhunthod, Xiaolong Zhou, Sarayut Tunmee, Suchinda Sattayaporn, Hui-Ming Cheng, Haiyan He, Yongbing Tang

**Affiliations:** 1 Functional Thin Films Research Center, Shenzhen Institutes of Advanced Technology, Chinese Academy of Sciences, Shenzhen, 518055 China; 2 Shenzhen College of Advanced Technology, University of Chinese Academy of Sciences, Shenzhen, 518055 China; 3 Synchrotron Light Research Institute, 111 University Avenue, Muang District, Nakhon Ratchasima, 30000 Thailand; 4 Tsinghua-Berkeley Shenzhen Institute, Tsinghua University, Shenzhen, 518055 China

**Keywords:** Batteries, Batteries, Batteries

## Abstract

Potassium-ion batteries are a compelling technology for large scale energy storage due to their low-cost and good rate performance. However, the development of potassium-ion batteries remains in its infancy, mainly hindered by the lack of suitable cathode materials. Here we show that a previously known frustrated magnet, KFeC_2_O_4_F, could serve as a stable cathode for potassium ion storage, delivering a discharge capacity of ~112 mAh g^−1^ at 0.2 A g^−1^ and 94% capacity retention after 2000 cycles. The unprecedented cycling stability is attributed to the rigid framework and the presence of three channels that allow for minimized volume fluctuation when Fe^2+^/Fe^3+^ redox reaction occurs. Further, pairing this KFeC_2_O_4_F cathode with a soft carbon anode yields a potassium-ion full cell with an energy density of ~235 Wh kg^−1^, impressive rate performance and negligible capacity decay within 200 cycles. This work sheds light on the development of low-cost and high-performance K-based energy storage devices.

## Abstract (web summary)

The abundance and low cost of potassium makes potassium batteries a promising technology for large scale energy storage. Here the authors apply a previously known frustrated magnet, KFeC_2_O_4_F, as the cathode in which the unique structure and Fe^2+^/Fe^3+^ redox enable excellent cycling stability.

## Introduction

Lithium-ion batteries (LIBs) are the state-of-the-art power technologies for portable electronic devices, electric vehicles, etc., owing to their high-energy density and long-term cycling life^1–6^. However, the limited resource of lithium has restricted some applications of LIBs, especially in the field of large-scale energy storage for intermittent renewable energies, such as solar, wind, and tidal energy^1,5^. Recently, rechargeable energy storage devices based on other alkali elements, such as sodium-ion batteries (SIBs)^7–12^ and potassium-ion batteries (KIBs)^13–16^, have attracted extensive attention because of their obvious advantage in abundant resources. Between them, KIBs have higher working voltages because of the lower redox potential of K/K^+^ (−2.93 V for K/K^+^, and −2.71 V for Na/Na^+^ vs SHE)^17^. In addition, K ion electrolyte exhibits a relatively higher conductivity due to the weaker Lewis acidity of K and the resultant smaller radii of solvated K ions, and the corresponding system displays a lower interfacial resistance caused by the smaller desolvation energy^18–20^. Although KIBs can utilize graphite-related materials as the anode to achieve a capacity of 280 mAh g^−1^ via the formation of KC_8_^21–24^, the development of high-performance KIBs is so far limited by the lack of appropriate cathode materials.

As the size of K^+^ (1.38 Å) is much larger than Na^+^ (1.02 Å) and Li^+^ (0.76 Å)^25^, large migration channels and stable crystal structures are essential for K ion migration, which raises a great challenge to develop suitable cathodes. Among the cathode materials reported for KIBs^26–47^, Prussian blue analogs (PBAs) have received most intensive studies with good capacity and favorable cycling stability because of their 3D open framework structures^14,31–35,48–50^. For instance, Goodenough et al. reported a cyanoperovskite K_x_MnFe(CN)_6_ (0 ≤ *x* ≤ 2) cathode for KIBs, delivering a stable discharge capacity of ~ 100 mAh g^−1^ and a capacity retention of 91% over 100 cycles^33^. More recently, Zhang et al. demonstrated RGO@PB@SSM cathode with a discharge capacity of 61.4 mAh g^−1^ and a lifespan of 305 cycles in KIBs^31^. Nevertheless, the preparation and handling of these materials are relatively difficult in controlling defects and water. The vacancies in the [TM(CN)_6_] (TM = Fe, Mn, etc.) framework and residual water from the synthesis process reduce the available electron reservoir, and lead to slow kinetics, low Coulombic efficiency (CE), and poor cycle life^24,28^. Further optimization of PBAs or searching for new cathode materials with a similar 3D framework is promising strategies to break through the limitation of cathode and pave the way to practical use of KIBs.

Upon close inspection of a magnetic material, KFeC_2_O_4_F, we notice that its 3D open structure is similar to PBAs^51,52^, which is very promising for K ion storage. Therefore, we carry out systematical electrochemical investigation of KFeC_2_O_4_F as a cathode material in KIBs using combined experimental and theoretical approaches. After optimization, a stable capacity of 112 mAh g^−1^ has been obtained and high-capacity retention of 94% is remained after 2000 cycles, with 0.003% capacity fading per cycle, which is the best long-term stability among the reported KIBs cathode materials. Moreover, a K-based full cell has been constructed by coupling this KFeC_2_O_4_F cathode with a soft carbon anode, which exhibits a reversible capacity of ~ 85 mAh g^−1^ (based on the mass of the cathode) and negligible capacity decay within 200 cycles, as well as impressive rate performance, paving the way for developing highly stable and low-cost K-based energy storage devices.

## Result

### Materials characterization

**Fig. 1:**
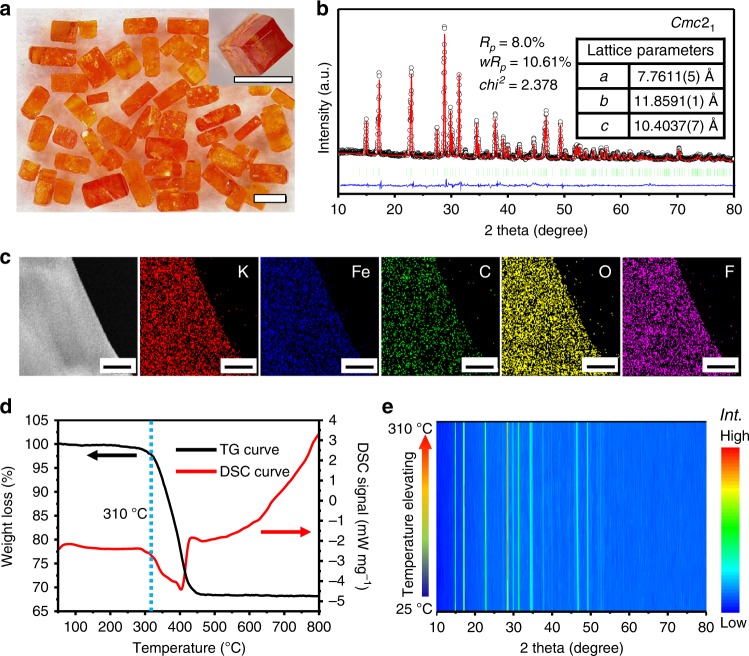
Characterization of KFeC_2_O_4_F. **a** Optical images of as-synthesized crystallites. The inset shows a tetra-prismatic single crystal in the size of ~ 1.0 × 0.6 × 0.5 mm. (scale bar = 1 mm) **b** Rietveld fitness of powder XRD (Cu *K*_α_) on a pristine sample. The inset shows the result of the fitness. **c** TEM and corresponding EDS mapping of K, Fe, C, O, F. (scale bar = 500 nm) **d** TG and DSC curves of a powder sample. **e** Variable–temperature XRD from 25 °C to 310 °C in air gas flow (Cu *K*_α_).

KFeC_2_O_4_F crystallites were synthesized via an optimized hydrothermal process (see Methods). An optical image is shown in Fig. 1a, and the inset shows a single crystallite, which exhibits a typical tetra-prismatic morphology in the size of ~ 1.0 × 0.6 × 0.5 mm. The phase of the prepared sample was checked by Rietveld refinement on powder X-ray diffraction (XRD) as illustrated in Fig. 1b. All diffraction peaks could be well indexed in the space group of *Cmc*2_1_ with the lattice parameters *a* *=* 7.7611(5) Å, *b* *=* 11.8591(1) Å, *c* *=* 10.4037(7) Å, in accordance with the previously reported data^52^. The Rietveld refinement also revealed that there is no detectable impurity in the sample. A sheet of KFeC_2_O_4_F (Supplementary Fig. 1) with the size of ~ 6 μm × 4 μm × 80 nm was prepared by focused ion beam and then examined by a transmission electron microscope (TEM) and energy dispersion spectroscopy (EDS), as shown in Fig. 1c. The results confirm the evenly distribution of K, Fe, C, O, and F in the obtained crystallites. The Fourier transform infrared and Raman spectra shown in Supplementary Fig. 2 confirm the existence of typical vibration peaks of the oxalate group (Supplementary Table 1)^53^. Meanwhile, our attempts in taking high-resolution TEM or selected area electron diffraction proved unsatisfactory owing to the sample’s sensitivity to intense electron beams, similar to some other oxalate, fluorosulphates and metal-organic frameworks.^54–57^

The thermal behavior of KFeC_2_O_4_F was analyzed by simultaneous thermogravimetric–differential scanning calorimetry and variable-temperature XRD. As shown in Fig. 1d, negligible weight loss was detected from the TG curve until 310 °C, confirming the good stability of KFeC_2_O_4_F below this temperature. A distinct weight loss step of *ca*. 32% from 310 °C to 450 °C was exhibited in the TG curve, accompanied by a broad endothermic peak in the DSC curve, which is mainly attributed to the decomposition of oxalate groups. Thereafter, XRD patterns at different temperatures were measured between room temperature and 310 °C (Fig. 1e), demonstrating the excellent phase stability in the chosen temperature range.

### Electrochemical performance as KIB cathode in half cells

To apply KFeC_2_O_4_F as a cathode material in KIBs, pristine KFeC_2_O_4_F was downsized and mixed with a carbon conductor by ball milling to improve its electric conductivity. The purity and morphology of samples after each treatment were checked by XRD, scanning electron microscope (SEM) and EDS element mapping of K, Fe, C, O, F (details in Supplementary Figs. 3, 4). After these treatments, the diffraction peaks of XRD patterns are consistent with those of the pristine sample, which indicates no phase change during ball milling. From SEM images, KFeC_2_O_4_F particles with an average size of *ϕ* ~ 250 nm were homogeneously mixed with the conductive carbon after ball milling, significantly smaller than hand-ground KFeC_2_O_4_F particles (Supplementary Fig. 5). This composite cathode was installed in coin-type cells to characterize the electrochemical performance of KFeC_2_O_4_F.

**Fig. 2:**
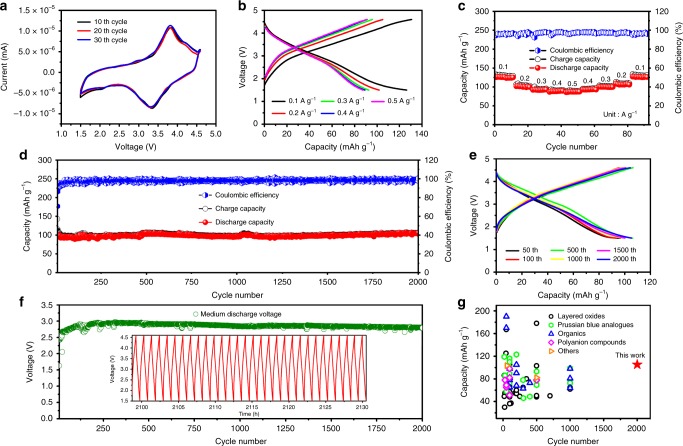
Electrochemical measurements of KFeC_2_O_4_F in K half-cells. **a** CV curves in different cycles at a scan rate of 0.1 mV s^−1^. **b** Galvanostatic charge–discharge curves at different current densities of 0.1–0.5 A g^−1^. **c** Rate performance under different current densities. **d** Cycling performance at 0.2 A g^−1^ for 2000 cycles, and **e** the corresponding charge-discharge curves of diverse cycles, and **f** medium discharge voltages. Inset of **f** is the charge–discharge curves for the final 30 cycles. **g** The comparison of cycling stability between our work and recently reported cathode materials (detailed in Supplementary Table 2) for potassium-ion batteries.

K half-cells were firstly fabricated in Ar-filled glove box, with K metal as anode and 1 m KPF_6_ in PC + EC (1:1 v/v) as the electrolyte. Typical cyclic voltammogram (CV) curves of the KFeC_2_O_4_F electrode in 10th, 20th, and 30th cycles in the range of 1.5–4.6 V were recorded at a scan rate of 0.1 mV s^−1^ (Fig. 2a). The overlapping of these curves indicated the good cyclic stability of the cathode. Meanwhile, a pair of peaks at 3.80/3.35 V is observed in the electrochemical processes during the de-intercalation/intercalation of K ions, corresponding to Fe^2+^/Fe^3+^ redox reactions. The rate performance of the cathode is plotted in Fig. 2b, c. Similar profiles of charge-discharge curves are observed, with moderate increase of discharge plateau from 0.5 to 0.1 A g^−1^ (Fig. 2b). In addition, the discharge capacity of KFeC_2_O_4_F cathode is 126, 102, 93, 89, and 87 mAh g^−1^ for 0.1, 0.2, 0.3, 0.4, and 0.5 A g^−1^, respectively, with the corresponding CEs over 95% (Fig. 2c). The discharge capacity is recovered to 126 mAh g^−1^ when the current density is changed back to 0.1 A g^−1^, demonstrating both good rate capability and good capacity retention of KFeC_2_O_4_F. Moreover, a half cell was measured by the galvanostatic intermittent titration technique (details in Supplementary Fig. 6), to reveal K ion diffusion kinetics indicated by chemical diffusion coefficients (*D*). The results show a variation of *D* values with depths of charge/discharge as shown in Supplementary Fig. 7. On the whole, the *D* values of 10^−10^–10^−12^ cm^2^ s^−1^ were at the similar level to that of Li^+^ in popular cathode materials (*D* of Li^+^ in LiFePO_4_ (10^−14^–10^−15^ cm^2^ s^−1^) and in LiMn_2_O_4_ (10^−11^–10^−9^ cm^2^ s^−1^))^58^. Thus, the good rate performance of KFeC_2_O_4_F was likely attributed to the fast K ion diffusion in the [FeC_2_O_4_F] framework.

The cycling performance of the KFeC_2_O_4_F cathode was illustrated in Fig. 2d. The battery with a KFeC_2_O_4_F cathode delivered a stable discharge capacity of ~ 112 mAh g^−1^ at 0.2 A g^−1^ after electrochemical activation in initial several cycles and kept a high capacity retention of 94% after 2000 cycles, with only 0.003% capacity fading per cycle. Noted that the voltage profiles of the first few cycles differ from the stabilized ones, possibly attributing to the reaction of conductive carbon wrapped on active material. To figure out the main underneath mechanism, we checked the voltage profile of the conductive carbon, and found out that the first charge–discharge profile of KFeC_2_O_4_F composite cathode (Supplementary Fig. 8a) resembles that of the conductive carbon (Supplementary Fig. 8c). As the active KFeC_2_O_4_F cathode had been mixed thoroughly with conductive carbon by ball milling, our KFeC_2_O_4_F particles had been wrapped entirely by the conductive carbon, so that the first charge–discharge process was mainly originated from the reaction of conductive carbon. As the process went on, KFeC_2_O_4_F particles became more and more exposed to the electrolyte, and the intrinsic electrochemical feature of KFeC_2_O_4_F became more and more dominating in following charge–discharge profiles. Although the cell based on the conductive carbon initially delivered a relatively high capacity, but its capacity faded gradually to ~30 mAh g^−1^ after 1000 cycles (Supplementary Fig. 8d). Considering the content of the carbon black in KFeC_2_O_4_F cathode material is 25%, its contribution to the whole capacity shall be ~ 7 mAh g^−1^, thus the obtained capacity on composite cathode material mainly comes from the KFeC_2_O_4_F compound. Stable discharge capacities of ~ 126 mAh g^−1^ and 80 mAh g^−1^ were obtained at 0.1 A g^−1^ and 0.5 A g^−1^, respectively, (Supplementary Fig. 9), with CE constantly above 96.5%. The galvanostatic charging–discharging (GCD) curves at the 1000th and 2000th cycles at 0.2 A g^−1^ match well with that of the 100th cycle (Fig. 2e). A stable medium discharging voltage (*V*_*m*_) of ~2.85 V of the cell is observed for 2000 cycles (Fig. 2f). These results demonstrate the outstanding cyclic stability of the cell throughout the testing period (around 2000 h). Further, to reveal the distinctive nature of the long-term reaction kinetics of KFeC_2_O_4_F cathode, electrochemical impedance spectroscopy (EIS) tests in different cycles were performed, with the Nyquist plots shown in Supplementary Fig. 10. Each plot comprises a depressed semicircle in the high-medium frequency region originated from the charge transfer resistance (*R*_*ct*_), and a sloping line in the low frequency region attributed to Warburg impedance. It is obviously seen that the *R*_*ct*_ value rises first and descends later in the first five cycles, probably owing to the generation of cathode electrolyte interphase films. The *R*_*ct*_ kept almost constant after the first 10 cycles, again revealing the good stability of the cell. Dozens of compounds have been thoroughly investigated so far as cathode materials for KIBs. Figure 2g shows the comparison of cycling stability between KFeC_2_O_4_F and the reported cathode materials for KIBs (detailed data refer to Supplementary Table 2), demonstrating that the cycling stability of this cathode is significantly better than that of reported KIB cathodes.

### Investigation of the reaction mechanism

**Fig. 3:**
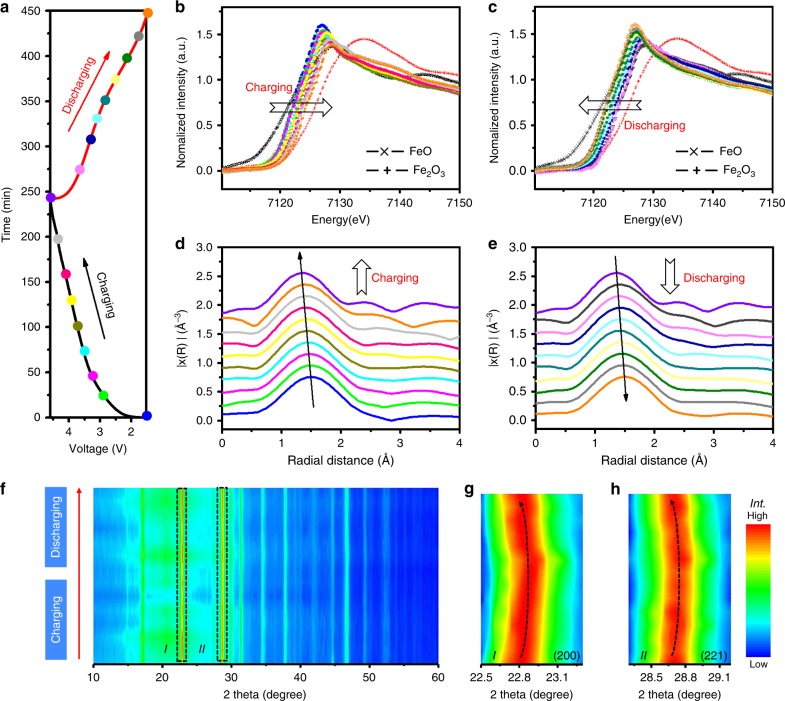
The structural evolution and charge compensation mechanism of the KFeC_2_O_4_F cathode. **a** A typical galvanometric charging–discharging curve of a stabilized half cell in the window of 1.5–4.6 V at a current density of 0.1 A g^−1^ and corresponding synchrotron Fe *K*-edge XANES during charging **b** and discharging **c**, and Fe EXAFS during charging **d** and discharging **e**. **f** Ex situ XRD of KFeC_2_O_4_F upon K ions extraction and reinsertion and the highlighted characteristic peaks in 22.5–23.3° corresponding to (200) plane **g** and 28.3–29.2° corresponding to (221) plane **h**.

To investigate the redox chemistry of KFeC_2_O_4_F as a cathode in KIBs, synchrotron-based in situ X-ray absorption spectra (in situ XAS) have been measured on a half cell during charging–discharging process, which is known as an advanced method to study the oxidation states and coordination environments of transition metals. The Fe absorption edge from 7000 to 7400 eV was recorded in a gas ionization chamber to track the incident and transmitted X-ray intensities in the transmission mode, whereas the GCD curve of the cell was recorded simultaneously in a battery testing system (see Methods). As a comparison, FeO and Fe_2_O_3_ were used for references. Figure 3a shows a typical GCD curve of the in situ cell during XAS measurement, and the color highlighted dots represent the states where in situ XAS were recorded. Figure 3b, c display the Fe *K*-edge X-ray absorption near-edge spectra (XANES) during charging and discharging, respectively, and Fig. 3d, e illustrate the corresponding extended synchrotron X-ray absorption fine-structure (EXAFS) spectra after* k*^*2*^-weighted Fourier transform. The Fe *K*-edge XANES spectra shown in Fig. 3b shifted to higher energy region during charging, which is ascribed to the transformation of Fe^2+^ to Fe^3+^ during K ion extraction. An opposite shift was observed during discharging (Fig. 3c), attributing to the reduction of Fe^3+^ to Fe^2+^ during K ion insertion. The change of XANES in a full cycle indicates a reversible change of Fe valence upon K ion extraction/insertion. In addition, the outline of spectra was mostly identical throughout a full cycle, indicating the high stability of the octahedral [FeO_4_F_2_] motif. In the analysis of EXAFS, oscillations within *k* = 2.6–10 Å^−1^ were chosen to minimize noise. As illustrated in Fig. 3d, e, broad peaks were identified at ~ 1.5 Å, corresponding to fourfold Fe-O and twofold Fe-F bonds in the octahedral [FeO_4_F_2_] motif. The peak shifted to lower distance upon charging, and vice versa upon discharging, suggesting Fe-O/F bonds contraction/expansion. The overlaps of XANES and EXAFS curves of the cathode at the beginning of the charging and the end of discharging (Supplementary Figs. 11, 12) also indicate the good reversibility of the process.

Apart from in situ synchrotron XAS, ex situ XRD patterns were also collected to explore the structural stability and the change of the lattice during K ion extraction/insertion. Figure 3f shows the XRD spectra of KFeC_2_O_4_F under different charging/discharging states. A broad peak centered at 20.5° was originated from the conductive carbon black (Supplementary Fig. 13a). It can be seen that the profiles of XRD patterns were almost unchanged within the cycle, indicating the good structural stability of the framework upon K ion extraction and insertion. The characteristic peaks of ex situ XRD in 22.5**–**23.3° and 28.3**–**29.2° are highlighted in Fig. 3g, h, corresponding to (200) and (221) plane, respectively. Both characteristic peaks display the same tendency of changes, that is, shifting to higher angle during charging and vice versa during discharging, corresponding to the lattice contraction and expansion, respectively. It should be noted that the shift is very small and reversible, indicating the finite volumetric change and high reversibility during the extraction/insertion of K ions. Moreover, a XRD pattern of KFeC_2_O_4_F cathode after long cycling was collected, indicating the well-maintained crystal structure after long cycling (Supplementary Fig. 13b).

### First-principle calculations

**Fig. 4:**
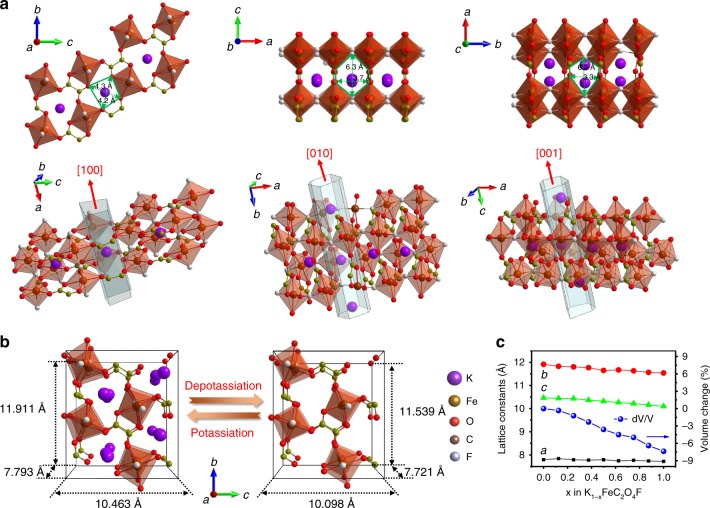
The structure of the KFeC_2_O_4_F cathode. **a** Projections of the KFeC_2_O_4_F structure along the [100], [010], and [001] directions and corresponding tunnels for possible ion migrations, with tunnel size highlighted. [FeO_4_F_2_] octahedra are represented in brown. **b** Unit cell of KFeC_2_O_4_F in potassiation/depotassiation states based on DFT calculation. **c** The lattice parameters and volume change of K_1–*x*_FeC_2_O_4_F as a function of the charge state *x*.

To illustrate the fundamental mechanism of the stable while reversible potassium storage in the framework of KFeC_2_O_4_F, in-depth structural analysis was performed, along with density functional theory (DFT) calculations based on the DFT + U method, in which the Hubbard U term stands for the strong onsite Coulomb repulsion of localized Fe 3*d* electrons. As illustrated in Fig. 4a, KFeC_2_O_4_F has a 2D [Fe(C_2_O_4_)]_∞_ network with F ions linking adjacent layers common, building a three dimensional [Fe(C_2_O_4_)F]_∞_ skeleton. Three tunnels along the [100], [010], and [001] directions can be delimited with the cross-section dimensions of (4.3 × 4.2 Å), (6.3 × 3.7 Å), and (6.3 × 3.3 Å), respectively. Such large tunnels are feasible for K ion migration. The DFT calculations results that the lattice parameters of KFeC_2_O_4_F transform from *a* = 7.793 Å, *b* = 11.911 Å, *c* = 10.463 Å, to *a* = 7.721 Å, *b* = 11.539 Å, *c* = 10.098 Å, after full extraction of K from KFeC_2_O_4_F to form FeC_2_O_4_F (Fig. 4b). This lattice change only results in 7.6% volume shrinkage, comparable to that of LiFePO_4_ (~ 7.8%)^59^. Moreover, the smooth evolution of lattice constants and volume change at varied charging stages (Fig. 4c) could reduce the possibility of mechanical failure (e.g., crack formation) during cycling.

**Fig. 5:**
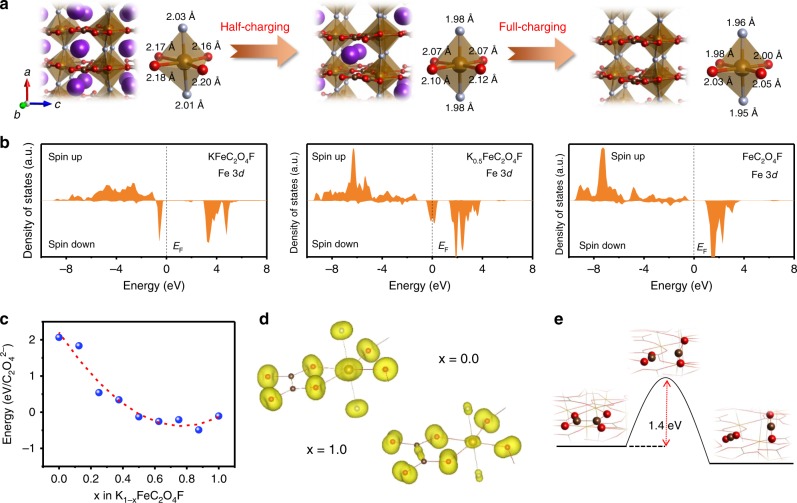
First-principles calculations. **a** Calculated atomic structures of K_1–*x*_FeC_2_O_4_F in the pristine, half-charging and full-charging states (*x* = 0, 0.5, 1), and **b** their corresponding partial density of states (DOS) projected onto the Fe 3*d* orbitals. **c** The reaction energy of a C_2_O_4_^2−^ decomposing into 2CO_2_ in the bulk of K_1–*x*_FeC_2_O_4_F as a function of the charge state *x*. **d** The partial charge density for electrons in the energy range between −2.5 eV and the Fermi level (0 eV) of the pristine (*x* = 0) and full-charging (*x* = 1) states. **e** The reaction scheme and kinetics of a C_2_O_4_^2−^ decomposing into 2CO_2_ in the bulk of K_0.125_FeC_2_O_4_F. Based on the Brønsted-Evans-Polanyi relation, the reaction barrier is linearly related to the reaction energy. Therefore, the reaction barrier in K_0.125_FeC_2_O_4_F with the lowest decomposition energy represents the minimum barrier in K_1–*x*_FeC_2_O_4_F.

Figure 5a presents the detailed structural evolution of KFeC_2_O_4_F at different charging stages based on DFT calculation, which reveals that the 3D framework undergoes a slight change with the marginal shrinking of octahedral [FeO_4_F_2_] building blocks during charge. More specifically, the Fe-O bonds on the *bc* plane shorten by ~ 0.16 Å upon full depotassiation, whereas the Fe-F bonds along the *a* axis undergo much less shrinkage by 0.06 Å, showing that the interlayer bridging F ions play a pivotal role in stabilizing the 3D framework. Along with the structural changes, Fig. 5b displays the corresponding density of states of the Fe 3*d* orbitals. In the pristine KFeC_2_O_4_F, Fe^2+^ exhibits a high-spin electron configuration *t*_2g_^4^*e*_g_^2^ (*S* = 2). It is inferred that the spin-down states (*t*_2g_) shall be redox active since they locate right below the Fermi level (*E*_F_). As potassium ions being extracted, these *t*_2g_ states of Fe^2+^ shift gradually above the *E*_F_ while emigrating electrons to surrounding anions. In contrast, its majority spin-up states shift further below the *E*_F_. In the full-charging [FeC_2_O_4_F], Fe^3+^ exhibits a half-filled electron configuration *t*_2g_^3^*e*_g_^2^ in the highest spin state (*S* = 5/2), obeying the Hund’s rule. This calculated Fe^2+^ to Fe^3+^ transformation agrees with the in situ XANES measurements during charging in Fig. 3b. Moreover, the high structural stability of KFeC_2_O_4_F upon charging/discharging under long cycles intimately associates with the above atomic and electronic properties. Figure 5c shows that the energy of C_2_O_4_^2−^ decomposition in the bulk K_1–*x*_FeC_2_O_4_F continually decreases with the extraction of potassium ions over *x* < 0.875, caused by the depletion of Fe 3*d* electrons and the accumulation of C 2*p* electrons near the *E*_F_ that are responsible for the redox reaction upon charging (Fig. 5d). At *x* ≥ 0.875, it reveals a turning point to prevent the decomposition energy becoming too negative upon full K extraction, owing to the stable 3*d* electron configuration with half-filled *t*_2g_ and *e*_g_ orbitals in FeC_2_O_4_F according to the Hund’s rule. Although the reaction energy indicates thermodynamically possible decomposition of C_2_O_4_^2−^ in the K_1-x_FeC_2_O_4_F during deep charge, its kinetic barrier of ~1.4 eV shown in Fig. 5e effectively inhibits the structure decomposition under ambient condition, resulting in a stable 3D framework for potassium battery chemistry.

### Electrochemical performance of a full cell

**Fig. 6:**
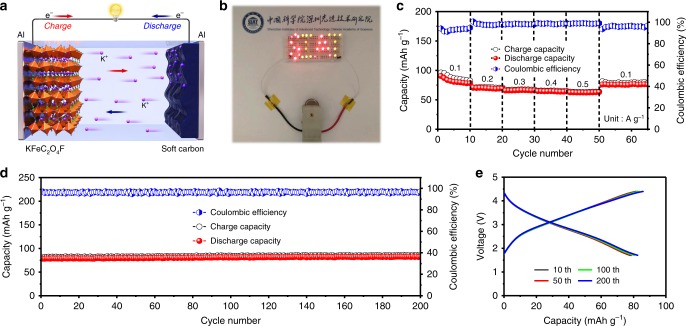
Electrochemical measurements of full K ion cell in 1.7–4.4 V. **a** Schematic illustration of working mechanism. **b** Lit LED screen driven by a K ion full cell. **c** Charge–discharge capacities and corresponding coulombic efficiency of a full cell under different current densities. **d** Cycling performance of a full cell at 0.1 A g^−1^, and **e** the corresponding charge–discharge curves of different cycles.

In practical applications, owing to the high combustibility of metallic K, a metallic K battery would not be safe. Following this consideration, a prototype K ion full cell was configured by combining KFeC_2_O_4_F cathode with a soft carbon (SC) anode. The working mechanism of the full cell is sketched in Fig. 6a, where K ions are removed from KFeC_2_O_4_F and migrate into the SC anode during charging, and a reverse transformation occurs during discharging. Figure 6b demonstrates that one prepared K ion full cell could power a LED screen. The rate performance of the full cell is shown in Fig. 6c, reaching a reversible capacity of 83.7, 71.0, 65.7 64.7, 62.6 mAh g^−1^ at a current rate from 0.1 to 0.5 A g^−1^. The discharge capacity returned back to 76.1 mAh g^−1^ when the current density recovered to 0.1 A g^−1^, demonstrating its good rate capability. Moreover, the full cell delivered a reversible capacity of ~ 85 mAh g^−1^ (based on the mass of the cathode) and remained stable with negligible capacity decay within 200 cycles at a current density of 0.1 A g^−1^ (Fig. 6d). The energy density of the full cell was estimated ~ 235 Wh kg^−1^. The GCD curves at 200th, 100th, 50th, and 10th cycles almost overlap with each other (Fig. 6e), manifesting the considerable cycling stability of our full battery.

## Discussion

In conclusion, we have identified KFeC_2_O_4_F as a promising cathode candidate for KIBs. Owing to its intrinsic Fe^2+^/Fe^3+^ redox mechanism, rigid 3D [FeC_2_O_4_F] framework and three open channels, the KFeC_2_O_4_F cathode yielded a reversible discharge capacity of ~ 112 mAh g^−1^ at 0.2 A g^−1^ and kept high capacity retention of 94% over 2000 cycles, with 0.003% capacity fading per cycle. Moreover, a full K ion cell was successfully constructed by combining this KFeC_2_O_4_F cathode with soft carbon anode, which delivered negligible capacity decay for 200 cycles with energy density of ~ 235 Wh kg^−1^ and impressive rate performance. Although the performance of this K ion full cell may be further enhanced by optimizing the electrolyte systems and utilizing suitable anode materials, etc., this study clearly indicates the feasibility of KFeC_2_O_4_F as a promising KIB cathode for sustainable large-scale energy storage applications.

## Methods

### Materials

Iron (II) oxalate dihydrate (FeC_2_O_4_∙2H_2_O, 99.5%), oxalic acid dihydrate (H_2_C_2_O_4_∙2H_2_O, 99.8%), potassium fluoroborate (KBF_4_, 99.99%), potassium carbonate (K_2_CO_3_, 99%), perylene-3,4,9,10-tetracarboxylic dianhydride (PTCDA), and potassium block (K, 99%) were purchased from Aladdin Reagent. Polyvinylidene fluoride (PVDF) binder, and *N*-methyl-2-pyrrolidone (NMP) were provided by Shenzhen KejingStar Technology. Ketjenblack EC600JD was purchased from Lion Corporation in Japan. Potassium hexafluorophosphate (KPF_6_, 99.99%), propylene carbonate (PC, 99.95%), ethylene carbonate (EC, 99.95%), and potassium molecular sieve were provided by Dodochem. The glass fiber separator (Whatman, 47 mm) was bought from Shanghai Huanao Technology Ltd. All of the above were used directly as received without further self-processing.

### Sample preparation

Single crystals of KFeC_2_O_4_F were grown via a hydrothermal reaction in mild conditions. A mixture of FeC_2_O_4_∙2H_2_O, H_2_C_2_O_4_∙2H_2_O, KBF_4_, and K_2_CO_3_ in the molar ratio of 1:3:2:4 (one for 1 mmol) was put in a Teflon-lined autoclave with 1 mL distilled water. After sealing, the autoclave was moved to a 180 °C oven for 48 h, then cooled to room temperature by turning off the oven. The resulting product was filtered with distilled water for several times until all by-products being removed, and then dried at a vacuum environment overnight. The soft carbon was fabricated facilely by thermal polymerizing PTCDA at a heating rate of 10 °C min^−1^ until 900 °C and kept for 4 h.

### Characterization

The optical images of as-synthesized crystallites were taken by a Leica DVM6M video microscope with ultra-depth-of-field. For the transmission electron microscope (TEM) characterization, a single crystallite was directly moved to the focused ion beam (FIB) chamber. A sheet section from the crystallite was prepared by a FEI Scios FIB/FE-SEM at an accelerating voltage of 30 kV under a selective etching process. The final sheet of the sample was positioned and secured on a Cu grid and transferred to the TEM chamber. The EDS of TEM were conducted by a JEOL JEM-3200FS field emission TEM equipped with an Oxford Instruments EDS analyzer. SEM measurements and EDS element mapping of the sample were carried out on ZEISS SUPRA 55 equipped with an EDS analyzer.

Powder XRD patterns were collected by a Rigaku diffractometer (MiniFlex600) with Cu *Kα* radiation (*λ* = 0.154056 nm). Scans were taken with a 2*θ* step of 0.02° over the range of 10–80°. Rietveld method was used to refine the data sets using the GSAS package incorporated with the EXPGUI interface^60^. Parameters, such as scale factor, background, lattice parameters, and zero point were refined until convergence. Analyses of recollected cathodes at different states, after being washed with PC to remove electrolyte and dried overnight, were conducted on the same XRD detector at a 2*θ* step of 2 ° min^−1^ over the range of 10–60°. Variable-temperature XRD patterns were recorded on a Smartlab 9KW diffractometer equipped with a SmartLab SOH-150F furnace with Cu *Kα* radiation. The scans were performed in the 2*θ* step of 2 ° min^−1^ in the range of 10–60° when heating samples from room temperature to 310 °C at various intervals.

The thermal behavior of KFeC_2_O_4_F was detected by simultaneous thermogravimetric and differential scanning calorimetry using a STA449F3 thermal analyzer (Netzsch, Germany). Hand-ground crystallites of ~ 10 mg were placed in an alumina crucible and heated from room temperature to 800 °C under a flow of air at a rate of 10 °C min^−1^. Fourier transform infrared (FTIR) spectrum was collected by a Perkin Elmer Frontier FTIR spectrometer in the range 400–4000 cm^−1^ with a resolution of 1 cm^−1^. The Raman spectrum was carried out using HORIBA, XploRA PLUS detector in the backscattering mode at the frequency range of 400–2000 cm^−1^. The wavelength resolution and probe aperture are 1 cm^−1^ and near 10 μm, respectively.

### Electrochemical characterization

The electrochemical performance of KFeC_2_O_4_F was studied in CR2032 coin-type half-cells assembled in an glove box (MIKROUNA) filled with high pure Argon gas, with water and oxygen levels < 0.1 ppm. KFeC_2_O_4_F crystallites were first hand-milled for several minutes and then ball milled for 4 h using a high-energy planetary ball mill. To improve the conductivity of KFeC_2_O_4_F, the above-mentioned sample was ball milled again with Kejten black carbon (3:1 w/w) for 8 h. Samples at each states were checked under powder XRD, ensuring the sample was not contaminated or destroyed during milling (Supplementary Figs. 3, 4). The subsequent powder was mixed with PVDF (85%:15% w/w), and NMP was added drop-by-drop until forming a homogeneous slurry. The slurry was then hand coated onto a carbon-coated Al foil, and dried at 80 °C in vacuum for 12 h and punched into circular sheets with diameters of 10 mm and loading mass of ~1.0 mg cm^−2^. Potassium metal foils, 1 m KPF_6_ in EC: PC = 1:1 (v/v) and glass fiber sheets (16 mm in diameter) were used as counter electrode, electrolyte and separators, respectively. Galvanostatic charge–discharge and galvanostatic intermittent titration technique (GITT) tests were performed on a battery test system (NEWARE CT-4008). Before GITT measurements, cells were initially activated for several cycles at 0.1 A g^−1^, and subsequently charged or discharged for 10 min at a pulse current of 50 mA g^−1^, followed by a duration of 10 min relaxation to achieve potassium equilibrium potential. Cyclic voltammetry and electrochemical impedance spectroscopy were performed on an Autolab (PGSTAT302N, Switzerland) electrochemical workstation. The full battery was assembled in similar conditions as half-cells, except that the anode was replaced by SC.

Synchrotron-based X-ray tests were performed on CR2016 coin-type half-cells with two sides kapton windows at SUT-NANOTEC-SLRI XAS beamline (BL5.2), Synchrotron Light Research Institute (SLRI, public organization), Thailand.^61^ A beam energy of 1.2 GeV generates the synchrotron radiation source at the storage ring and the beamline photon source covers an energy range of 40–1040 eV at the resolving power of 10,000. The typical loading of KFeC_2_O_4_F cathode is 5–8 mg cm^−2^.

### Calculations

All calculations presented were performed by the Vienna ab initio simulation package based on spin-polarized DFT^62,63^, with projector-augmented-wave method^64,65^ and a plan-wave cutoff energy of 450 eV. The Perdew–Burke–Ernzerhof functional^66^ with the Hubbard U correction^67^ was adopted for the exchange correlation energy. A value of 4.0 eV was applied to U to correct the onsite Coulomb repulsion of Fe *3d* electrons^68^. The Brillouin zone was sampled with a 6 × 4 × 4 Γ-centered k mesh to keep the reciprocal spacing of all calculations with supercells of K_8–*x*_Fe_8_(C_2_O_4_)_8_F_8_ (8 ≥ *x* ≥ 0) < 0.03 Å^−1^. The criteria for energy convergence was set to be 10^−5^ eV. The optimized orthorhombic lattice of K_8_Fe_8_(C_2_O_4_)_8_F_8_ is 7.81 × 11.92 × 10.48 Å, consistent well with the lattice determined by XRD as 7.76 × 11.86 × 10.40 Å. The oxalate dissociation barriers in the bulk were simulated using climbing image nudged elastic band method^69^ with force convergence criteria on each atom of 0.05 eV Å^−1^.

## Supplementary information

Supplementary Information

## Data Availability

The data that support the plots within this paper and other findings of this study are available from the corresponding author upon reasonable request.
