# Supplementary material for: A fluoroxalate cathode material for potassium-ion batteries with ultra-long cyclability
Source: Nat Commun. 2020 Mar 6;11:1225. doi: 10.1038/s41467-020-15044-y (PMC7060185; doi:10.1038/s41467-020-15044-y)
Supplement: Supplementary file 1 — Supplementary Information [file 41467_2020_15044_MOESM1_ESM.pdf]

Supplementary Information for

A fluoroxalate cathode material for potassium-ion batteries with ultra-long cyclability

Ji et al.

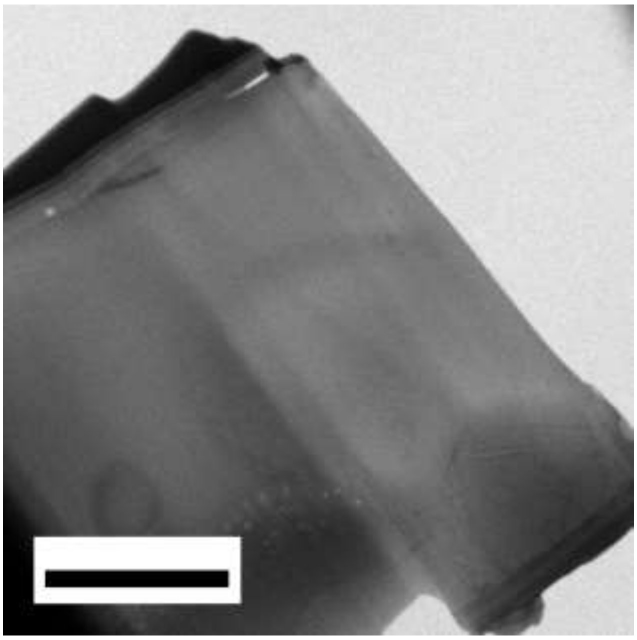

Supplementary Figure 1 | TEM image of a $KFeC_2O_4F$ sheet prepared by focused ion beam (scale bar = 2 µm).

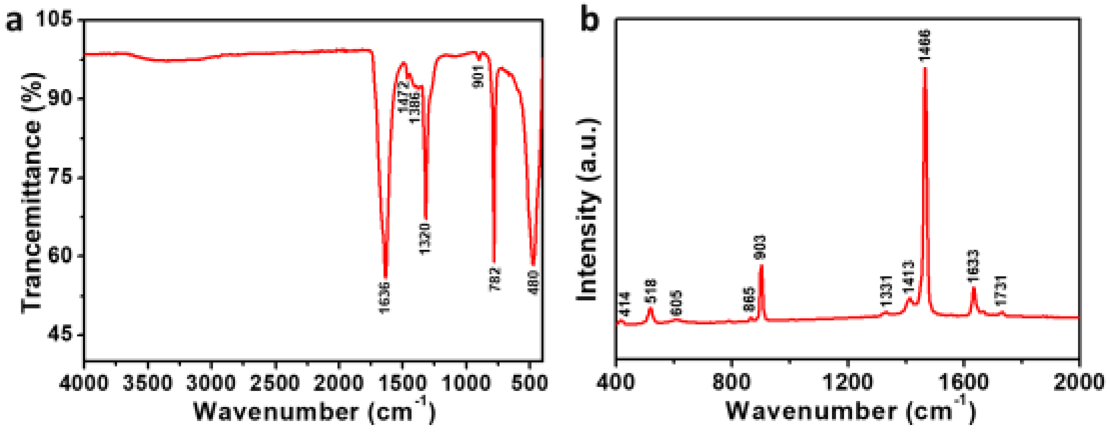

Supplementary Figure 2 | Spectra of pure $KFeC_2O_4F$ crystallites. a, Fourier transform infrared spectrum in the range of 400–4000 cm^{-1}. **b**, Raman spectrum in the range of 400–2000 cm^{-1}.

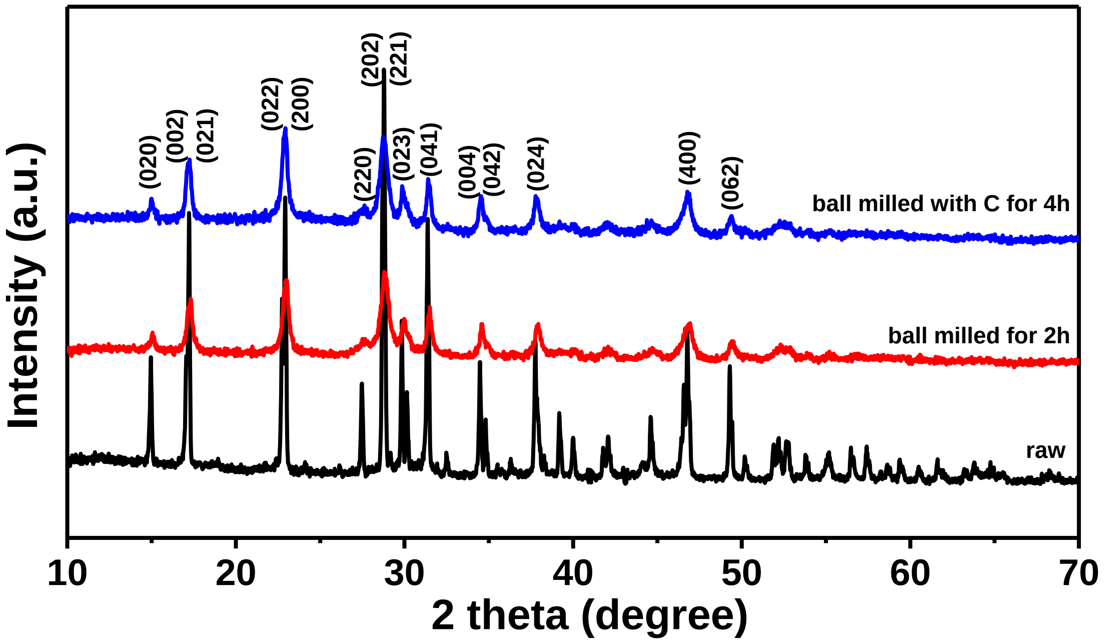

Supplementary Figure 3 | XRD profiles of the raw sample, the sample ball milled and the sample ball milled with conductive carbon.

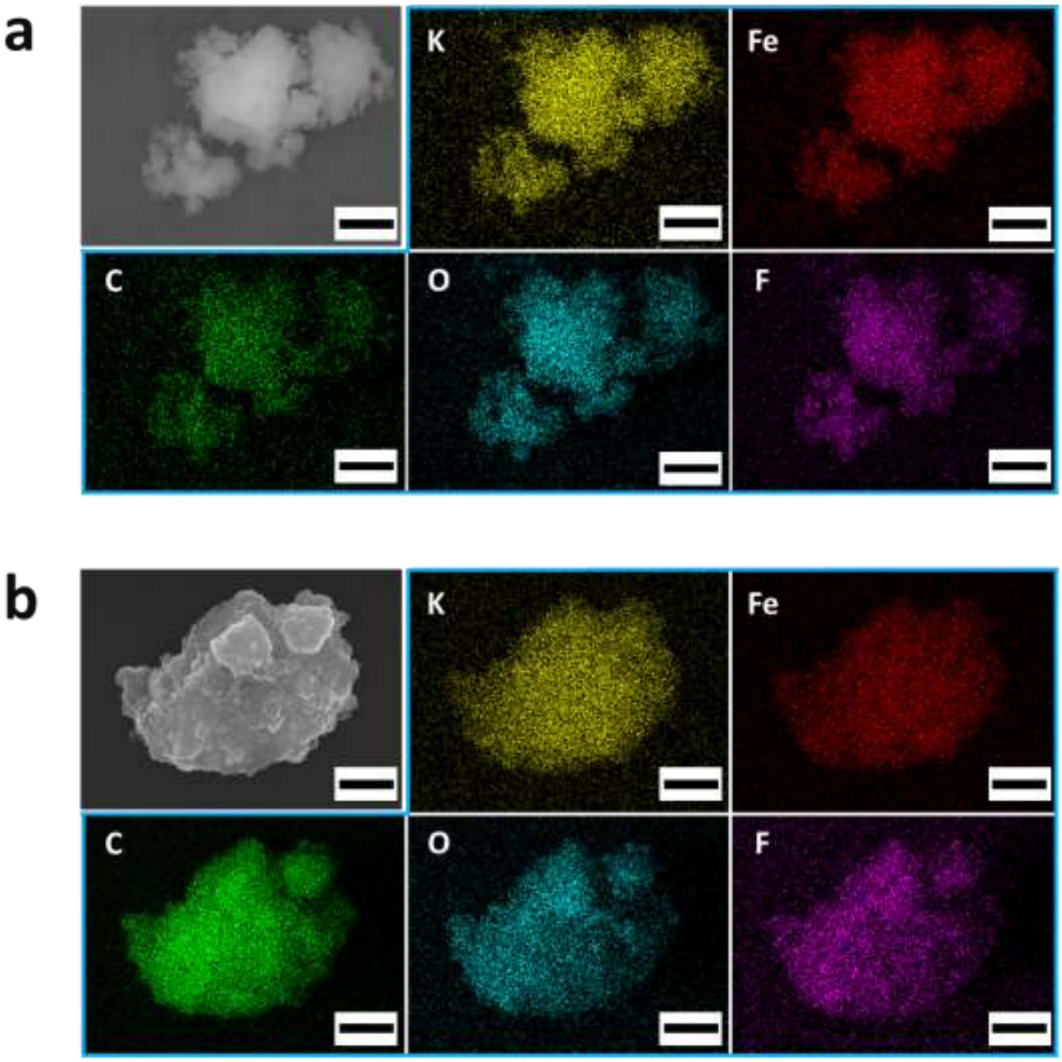

Supplementary Figure 4 | Scanning electron microscope images and energy dispersion spectra element mapping of K, Fe, C, O, F. a, Sample after ball-milling. **b**, Sample ball milled with conductive carbon (scale bar = 2 μm).

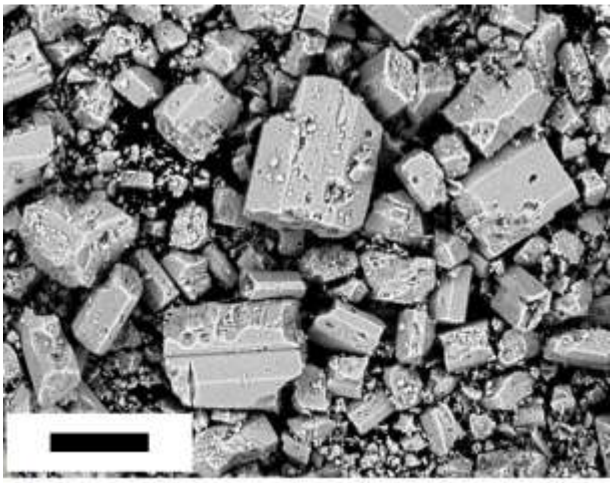

Supplementary Figure 5 | Scanning electron microscope image of a hand-ground $KFeC_2O_4F$ sample (scale bar = 40 µm).

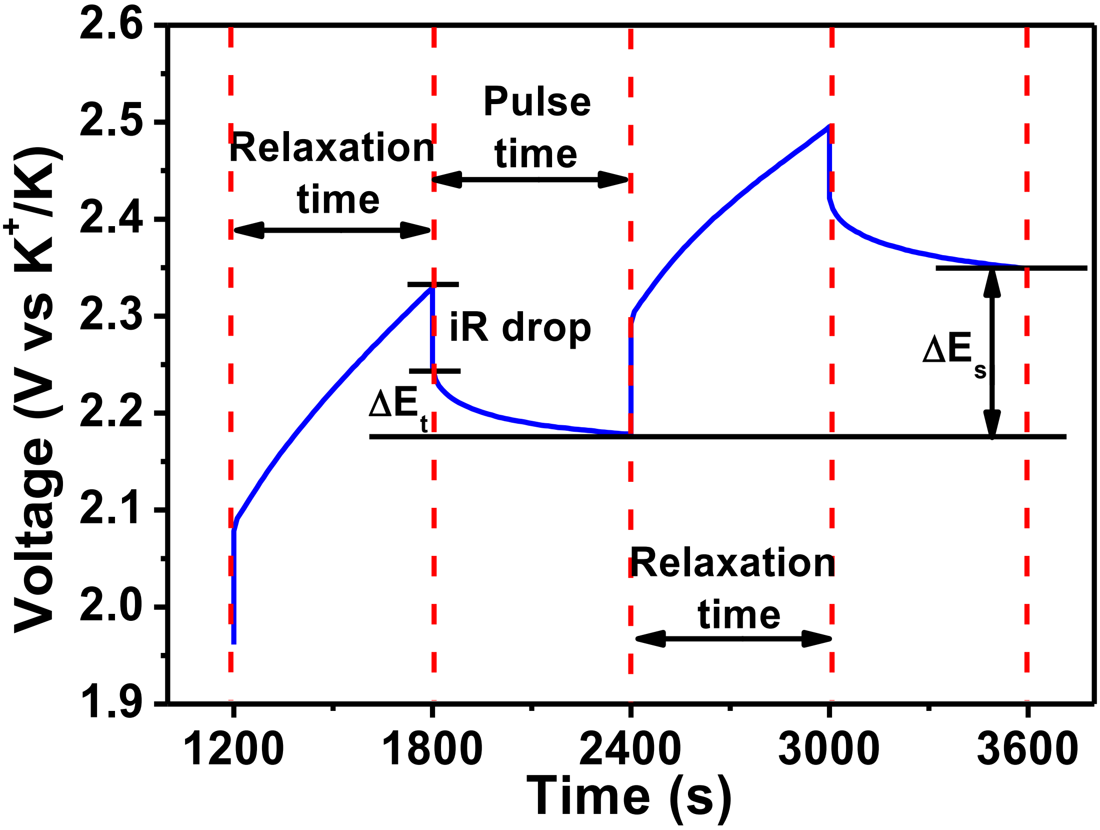

Supplementary Figure 6 | *E* vs. *t* curves of $KFeC_2O_4F$ electrode for a single GITT during discharge process.

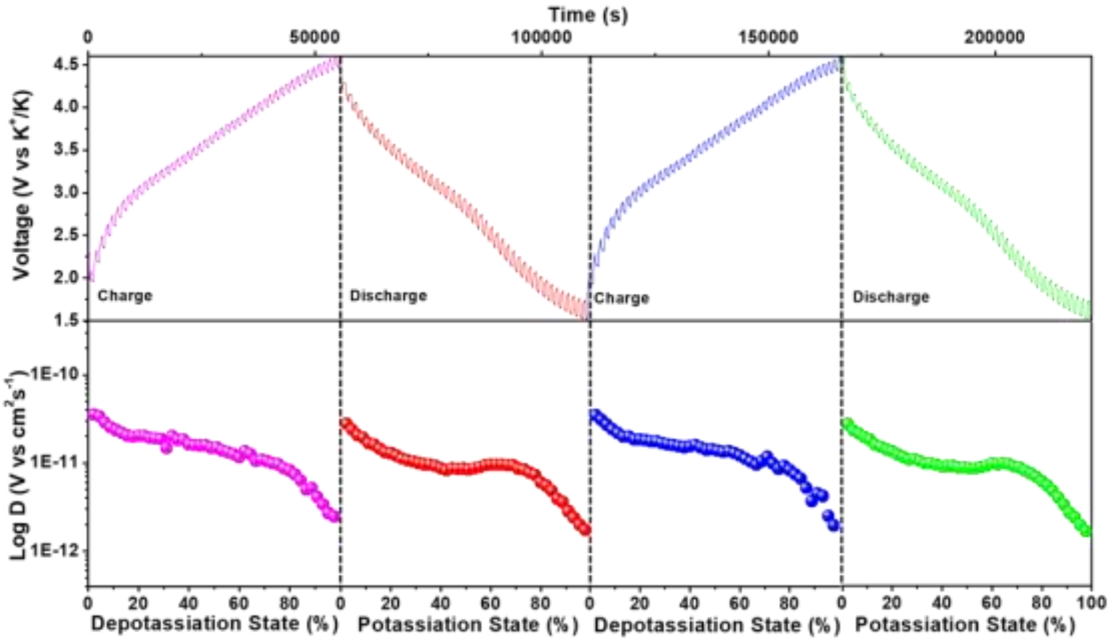

Supplementary Figure 7 | GITT curves and the corresponding K ion diffusion coefficient at different discharge/charge state of $KFeC_2O_4F$ cathode.

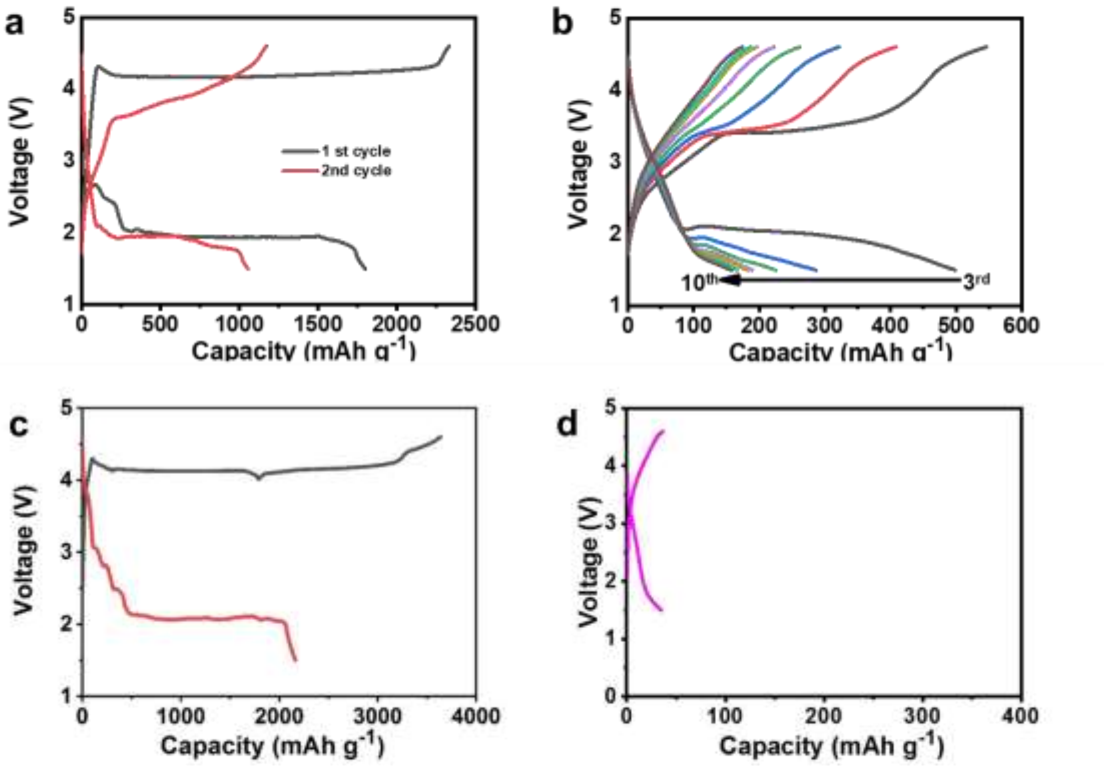

Supplementary Figure 8 | Charge-discharge profile of active material and conductive carbon in different cycles. a, first two cycles of $KFeC_2O_4F$-based half-cell. **b,** third to 10th cycles of $KFeC_2O_4F$-based half-cell. **c,** first cycle of conductive carbon. **d,** 1000th cycle of conductive carbon. Due to the high specific area of conductive carbon and high applied voltage, the side reaction of the conductive carbon is tremendous in the first cycle. Besides, since the $KFeC_2O_4F$ particles had been wrapped thoroughly by conductive carbon, it is seen that the first cycle of $KFeC_2O_4F$-based half-cell resembles that of the conductive carbon a lot.

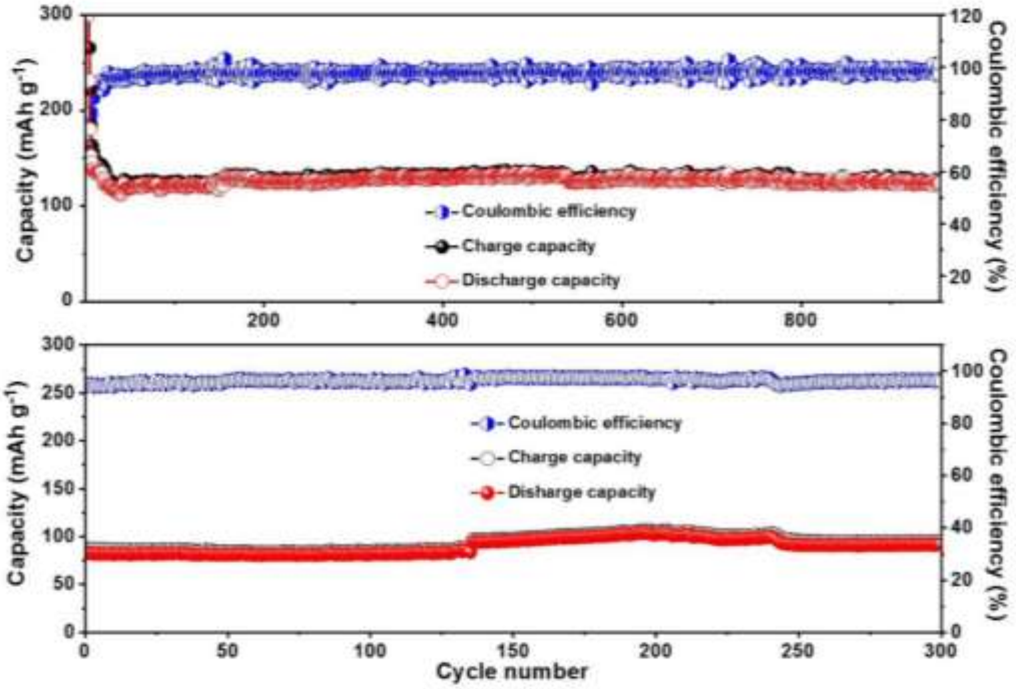

Supplementary Figure 9 | Cycling performance of a half cell at 0.1 A g^{-1} (up) and 0.5 A g^{-1} (bottom).

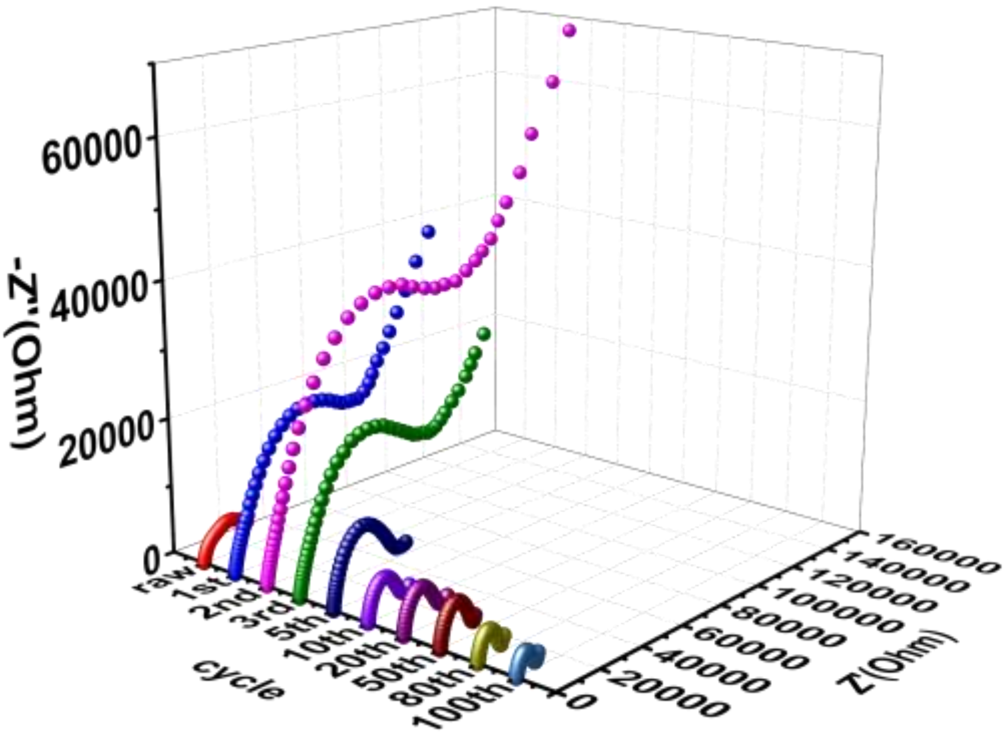

Supplementary Figure 10 | Nyquist plots at different cycles.

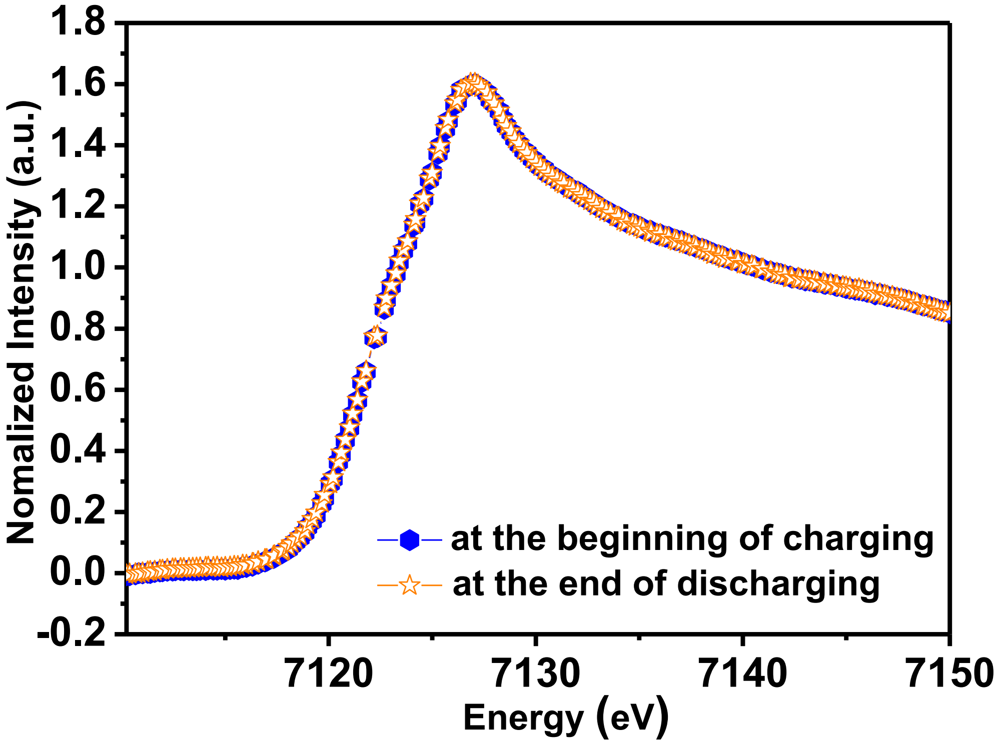

Supplementary Figure 11 | Synchrotron Fe *K*-edge XANES of $KFeC_2O_4F$ at the beginning of charging and the end of discharging. The super-impose of the two profiles indicates the good reversibility of the cycling.

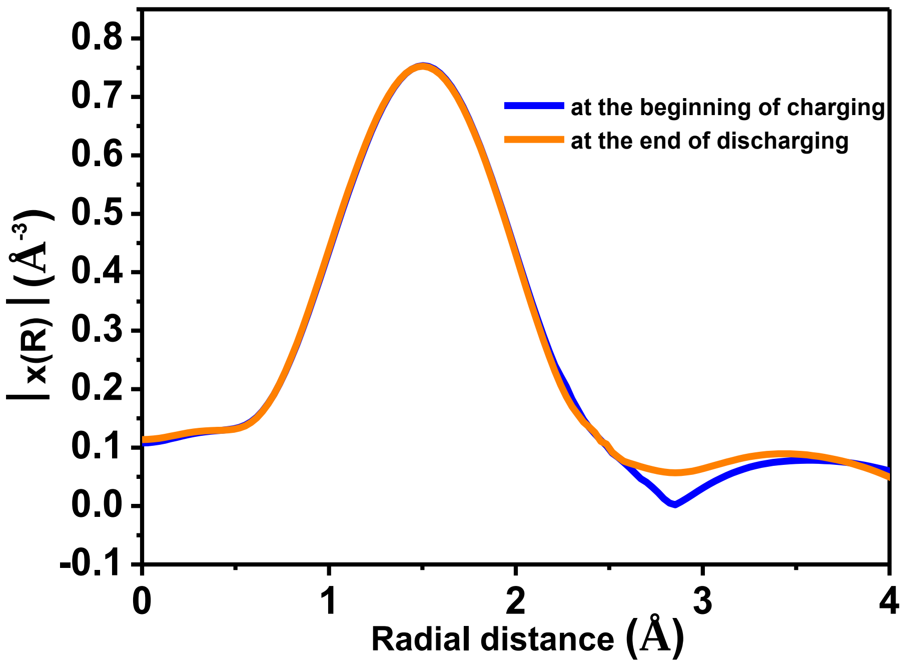

Supplementary Figure 12 | Synchrotron Fe EXAFS of $KFeC_2O_4F$ at the beginning of charging and the end of discharging.

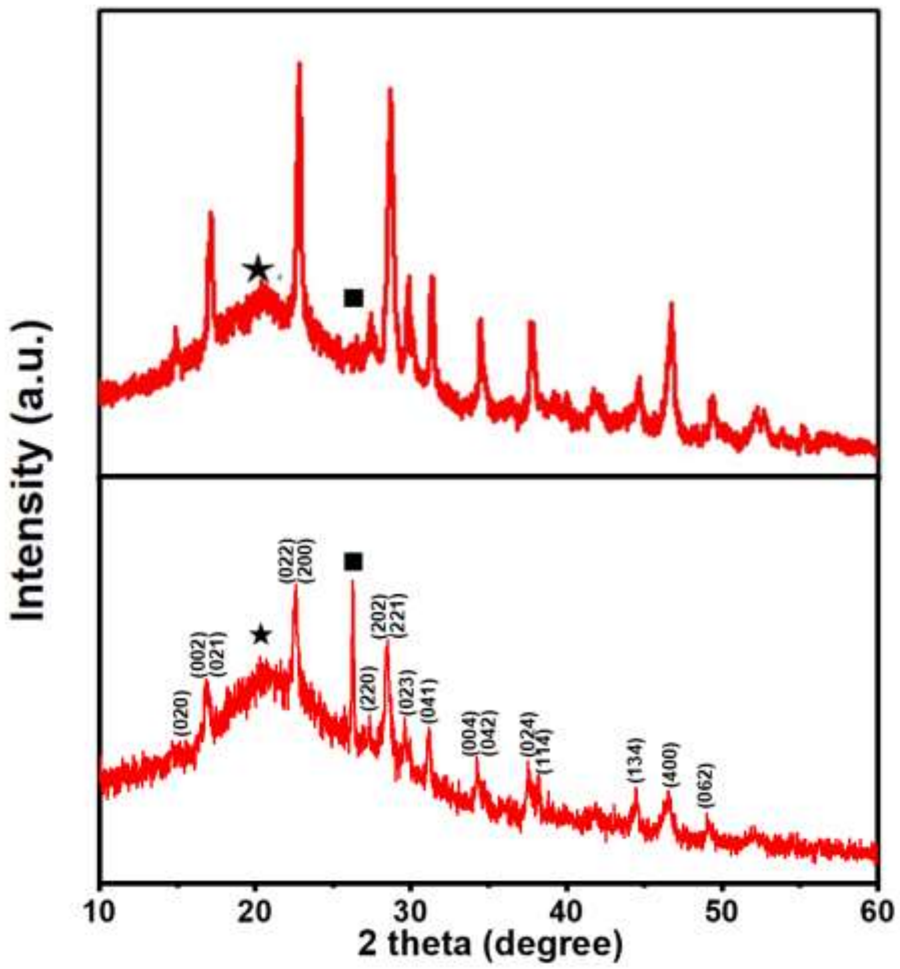

Supplementary Figure 13 | XRD profile $KFeC_2O_4F$ electrode. Up: initial casting electrode; bottom: recollected electrode after 500 cycles. The star and rectangular peak originated from conductive carbon and current collector, respectively.

Supplementary Table 1 | Assignments of FTIR and Raman spectra of pure $KFeC_2O_4F$.

(Corresponding to Supplementary Figure 2)

IR	Raman	Band assignments
1636		ν_{as} (C=O)
1472		ν_s (C-O) + ν(C-C)
1386		ν_s (C-O) + δ(O-C=O)
1320		ν_s (C-O) + δ(O-C=O)
901		ν_s (C-O) + δ(O-C=O)
782		δ(O-C=O) + ν(M-O)
480		Ring deform + δ(O-C=O)
	1731	ν (CO)
	1633	ν (CO)
	1466	ν (C=O) stretching
	1413	ν (C=O) stretching
	1331	B_{2g}(O-C=O)
	903	ν (C-C) stretching
	865	δ(O-C=O) bending
	605	δ(O-C=O) bending
	518	ν (MO ring)
	414	δ(MO ring)

Supplementary Table 2 | Electrochemical performances of $KFeC_2O_4F$ and cathode materials of recently reported for potassium-ion batteries.

Cathode material	Type	Voltage [V]	Current density (mA g^{-1})	Stable capacity	Cycle number	Capacity after cycles (mA g^{-1})	Ref.
P2-type $K_{2/3}Ni_{1/3}Co_{1/3}Te_{1/3}O_2$	Layered	1.25–5.0	6.5	30	25	30	1
P2-Type $K_{0.65}Fe_{0.5}Mn_{0.5}O_2$	Layered	1.5–4.2	100	103	350	80	2
P2-type $Na_{0.84-x}K_xCoO_2$	Layered	2.0–4.2	7.6	82	50	69	3
P2-type $K_2Ni_2TeO_6$	Layered	1.3–4.7	6	70	70	62	4
P2-Type $K_{0.6}CoO_2$	Layered	1.7–4.0	100	62	120	37	5
P2-type $K_{0.6}CoO_2$	Layered	1.7−4.0	40	75	300	65	6
P3-type $K_{0.45}MnO_2$	Layered	1.5−4.0	20	101	100	66.7	7
P′2-type $K_{0.3}MnO_2$	Layered	1.5–3.5	28	74	700	50	8
P2-type $K_{0.41}CoO_2$	Layered	2.0–3.9	11.8	57	30	54	9
P3-type $K_{2/3}CoO_2$	Layered	2.0–3.9	11.8	60	30	55	9
P3-$K_{0.69}CrO_2$	Layered	1.5–3.8	100	100	1000	65	10
P3-Type $K_{0.5}MnO_2$	Layered	1.5–4.2	5	140	20	49	11
P3-Type $K_{0.5}MnO_2$	Layered	1.5–3.9	5	110	20	86	11
P3-type $K_{0.45}MnO_2$	Layered	1.5–4.0	20	101	100	71.5	7
P3-$K_{0.54}[Co_{0.5}Mn_{0.5}]O_2$	Layered	1.5–4.0	500	78	500	48	12
P′3-type $Na_{0.52}CrO_2$	Layered	2.0–3.6	500	52	200	51	13
O3-type $KCrS_2$	Layered	1.8–3.0	9	71	1000	64	14
O3-type $KCrO_2$	Layered	1.5–4.0	10	85	100	57	15
$K_{0.37}Na_{0.3}Ni_{0.17}Co_{0.17}Mn_{0.66}O_2$	Layered	2.0–4.2	20	86.1	100	78.8	16
$K_{0.67}Ni_{0.17}Co_{0.17}Mn_{0.66}O_2$	Layered	2.0–4.3	20	80	100	72	17
$K_2[(VO)_2(HPO_4)_2(C_2O_4)]$	Layered	2.0–4.6	22	65	200	54	18
$K_{0.7}Fe_{0.5}Mn_{0.5}O_2$	Layered	1.5–4.0	20	178	45	125	19
$K_{1.06}Mn_8O_{16}$/CNT	Layered	1.5–4.0	500	125	100	72.2	20
$V_2O_5\cdot 0.6H_2O$	Layered	1.5–4.0	50	155	500	103	21
$K_xV_2O_5\cdot nH_2O$	Layered	2.0–4.3	20	226	50	167	22
$K_{0.77}MnO_2\cdot 0.23H_2O$	Layered	1.5–4.0	1000	77	1000	62	23
$K_{0.32}MnO_2$	Layered	2.0–4.5	100	49.2	100	36.1	24
$Na_{0.9}Cr_{0.9}Ru_{0.1}O_2$	Layered	1.5–3.8	500	62	500	50.3	25
$VOPO_4\cdot 2H_2O$	Layered	-0.2–1.3 (vs Ag/AgCl)	27	88.3	100	76	26
$K_xMnO_2\cdot nH_2O$	Layered	1.5–4.0	80	86	50	68	27
AlF_3@S-KMO	Layered	1.5–4.0	10	110	100	105	28
$K_{0.5}V_2O_5$	Layered	1.0–3.0	100	60	250	49	29
K2V3O8/C	Layered	1.0–4.2	20	75	200	60	30
V_2O_5	Layered	2.0–4.5	100	77.8	100	48	31
V_2O_5@rGO	Layered	1.5–4.3	147	222	500	178	32
$K_{0.3}Ti_{0.75}Fe_{0.25}[Fe(CN)_6]_{0.95}\cdot 2.8H_2O$	PBAs	1.0–4.5	100	113	100	73.1	33
$K_{0.220}Fe[Fe(CN)_6]_{0.805}\cdot 4.01H_2O$	PBAs	2.0–4.0	50	74.5	50	73.2	34
$K_{1.6}Mn[Fe(CN)_6]_{0.96}\cdot 0.27H_2O$	PBAs	3.2–4.3	50	110	30	86	35
$K_{1.70}Mn[Fe(CN)_6]_{0.90}\cdot 1.10H_2O$	PBAs	2.5–4.6	156	110	100	100	36
$K_{1.75}Mn[Fe(CN_6)]_{0.93}\cdot 0.16H_2O$	PBAs	2.0–4.5	30	120	100	116	37
$K_{1.64}Fe[Fe(CN)_6]_{0.89}\cdot 0.15H_2O$	PBAs	2.0–4.5	30	122	100	110	37
$K_{1.88}Zn_{2.88}[Fe(CN)_6]_2(H_2O)_5$	PBAs	3.4−4.15	14	55.6	100	52.8	38
$K_{1.92}Fe[Fe(CN)_6]_{0.94}\cdot 0.5H_2O$	PBAs	2.0–4.3	13	133	200	123	39
$K_{1.7}Fe[Fe(CN)_6]_{0.9}$	PBAs	2.0–4.5	100	120	300	78	40
$K_{1.59}Fe_{2.20}(CN)_6$	PBAs	2.25–4.25	77.5	148	1000	99	41
$KFe[Fe(CN)_6]$	PBAs	2.0–4.5	100	90.7	1000	73	42
$KFe[Fe(CN)_6]$	PBAs	0.8–2.1 (vs Al)	2000	72	50	58	43
$KFe^{3+}[Fe^{2+}(CN)_6]$	PBAs	2.6–4.1	8.7	78	500	69	44
RGO@PB@SSM	PBAs	2.0–4.0	50	61.4	305	46	45
KHCF@PPy	PBAs	2.0–4.2	50	88.8	500	77.1	46
$K_2FeFe(CN)_6$	PBAs	2.0–4.3	20	110	100	89	47
$FeFe(CN)_6$	PBAs	0.0–0.975 (vs Ag/AgCl)	111	140	20	119	48
$K_4Fe(CN)_6$	PBAs	2.0–3.8	20	65.5	400	48.8	49

$FeFe(CN)_6$	PBAs	1.5–4.0	625	100	500	93	50
o-$Na_2C_6H_2O_6$	Organic	1.0–3.0	25	98.8	100	65.1	51
p-$Na_2C_6H_2O_6$	Organic	1.0–3.0	25	228.5	50	190	52
CuTCNQ	Organic	2.0–4.0	50	206	50	170	53
PTCDA	Organic	1.5–3.5	50	117	200	90	54
PTCDA	Organic	1.2–3.2	10	87	300	63	55
PTCDA	Organic	1.5–3.5	1000	113	1000	98	56
AQDS	Organic	1.4–3.0	13	95	100	78	57
AQDS	Organic	1.4–3.0	390	80	1000	64	58
PAQS	Organic	1.5–3.4	200	106	200	68	59
PTPAn	Organic	2.0–4.0	100	75	60	71	60
PVK	Organic	2.0–4.7	500	104	400	73	61
PAN	Organic	2.0–4.0	50	100	100	98	62
SR	Organic	1.5–3.8	125	118	100	82	63
PQ-1, 5	Organic	1.2–3.2 V	250	115	200	105	64
PI-CMP	Organic	1.5–3.5 V	1000	109	1000	80.8	65
$K_3V_2(PO_4)_3$/C	Polyanion	2.5–4.3	20	55	100	52	66
$KTi_2(PO_4)_3$/C	Polyanion	1.2–2.8	64	75.6	100	82	67
$KVPO_4F$	Polyanion	2.0–4.8	6.65	72	50	65	68
$KVOPO_4$	Polyanion	2.0–4.8	6.65	73	50	69	68
$KVPO_4F$	Polyanion	3.0–5.0	5	100	30	78	69
KVP_2O_7	Polyanion	2.0–5.0	25	60	100	51	70
$K_3V_2(PO_4)_3$	Polyanion	0.01–3.0 (vs KVP/C)	25	88	500	78	71
$K_3V_2(PO_4)_2F_3$	Polyanion	2.0–4.5	10	101	100	98	72
K(Mn,Co)F3	Others	1.2–4.2	40	132.6	60	103.4	73
I_2	Others	1.9–3.5 V	100	115	500	82	74
**$KFeC_2O_4F$**	**Polyanion**	**1.5–4.6**	**200**	**112**	**2000**	**105**	**This work**

Supplementary Methods

Calculation of potassium chemical diffusion coefficients (*D*)

The potassium chemical diffusion coefficients (*D*) were measured by using galvanostatic intermittent titration technique (GITT) and calculated based on Supplementary Eq.1 as follows:

$$\boldsymbol{D} = \frac{4}{\pi\tau}\left(\frac{V_m n_m}{S}\right)^2\left(\frac{\Delta E_s}{\Delta E_t}\right)^2 \qquad (1)$$

Where t is the duration of the current pulse (s), τ is the relaxation time (s), and ΔE_s is the steady-state potential change (V) by the current pulse. ΔE_t is the potential change (V) during the constant current pulse after eliminating the iR drop. n_m, V_m, and S are the number of moles (mol), molar volume of $KFeC_2O_4F$ (cm^3 mol^{-1}), and the surface area (cm^2), respectively.

Supplementary References:

1. Masese, T. *et al.* A high voltage honeycomb layered cathode framework for rechargeable potassium-ion battery: P2-type $K_{2/3}Ni_{1/3}Co_{1/3}Te_{1/3}O_2$. *Chem. Commun.* **55**, 985-988 (2019).
2. Deng, T. *et al.* Layered P2-Type $K_{0.65}Fe_{0.5}Mn_{0.5}O_2$ Microspheres as Superior Cathode for High-Energy Potassium-Ion Batteries. *Adv. Funct. Mater.* **28**, 1800219 (2018).
3. Sada, K., Senthilkumar, B. & Barpanda, P. Electrochemical potassium-ion intercalation in Na_xCoO_2: a novel cathode material for potassium-ion batteries. *Chem. Commun.* **53**, 8588-8591 (2017).
4. Masese, T. *et al.* Rechargeable potassium-ion batteries with honeycomb-layered tellurates as high voltage cathodes and fast potassium-ion conductors. *Nat. Commun.* **9**, 3823 (2018).
5. Kim, H. *et al.* K-Ion Batteries Based on a P2-Type $K_{0.6}CoO_2$ Cathode. *Adv. Energy Mater.* **7**, 1700098 (2017).
6. Deng, T. *et al.* Self-Templated Formation of P2-type $K_{0.6}CoO_2$ Microspheres for High Reversible Potassium-Ion Batteries. *Nano Lett.* **18**, 1522-1529 (2018).
7. Liu, C.-L., Luo, S.-H., Huang, H.-B., Zhai, Y.-C. & Wang, Z.-W. Layered potassium-deficient P2- and P3-type cathode materials K_xMnO_2 for K-ion batteries. *Chem. Eng. J.* **356**, 53-59 (2019).
8. Vaalma, C., Giffin, G. A., Buchholz, D. & Passerini, S. Non-Aqueous K-Ion Battery Based on Layered $K_{0.3}MnO_2$ and Hard Carbon/Carbon Black. *J. Electrochem. Soc.* **163**, A1295-A1299 (2016).
9. Hironaka, Y., Kubota, K. & Komaba, S. P2- and P3-K_xCoO_2 as an electrochemical potassium intercalation host. *Chem. Commun.* **53**, 3693-3696 (2017).
10. Hwang, J.-Y., Kim, J., Yu, T.-Y., Myung, S.-T. & Sun, Y.-K. Development of P3-$K_{0.69}CrO_2$ as an ultra-high-performance cathode material for K-ion batteries. *Energy Environ. Sci.* **11**, 2821-2827 (2018).
11. Kim, H. *et al.* Investigation of Potassium Storage in Layered P3-Type $K_{0.5}MnO_2$ Cathode. *Adv. Mater.* **29** 1702480 (2017).
12. Choi, J. *et al.* $K_{0.54}[Co_{0.5}Mn_{0.5}]O_2$: New cathode with high power capability for potassium-ion batteries. *Nano Energy* **61**, 284-294 (2019).
13. Naveen, N. *et al.* Reversible K^+-Insertion/Deinsertion and Concomitant Na^+-Redistribution in P′3-$Na_{0.52}CrO_2$ for High-Performance Potassium-Ion Battery Cathodes. *Chem. Mater.* **30**, 2049-2057 (2018).
14. Naveen, N. *et al.* $KCrS_2$ Cathode with Considerable Cyclability and High Rate Performance: The First K^+ Stoichiometric Layered Compound for Potassium-Ion Batteries. *Small* **14**, 1803495 (2018).
15. Kim, H. *et al.* Stoichiometric Layered Potassium Transition Metal Oxide for Rechargeable Potassium Batteries. *Chem. Mater.* **30**, 6532-6539 (2018).
16. Liu, C.-L., Luo, S.-H., Huang, H.-B., Zhai, Y.-C. & Wang, Z.-W. Influence of Na-substitution on the structure and electrochemical properties of layered oxides $K_{0.67}Ni_{0.17}Co_{0.17}Mn_{0.66}O_2$ cathode materials. *Electrochim. Acta* **286**, 114-122 (2018).
17. Liu, C. *et al.* $K_{0.67}Ni_{0.17}Co_{0.17}Mn_{0.66}O_2$: A cathode material for potassium-ion battery. *Electrochem. Commun.* **82**, 150-154 (2017).
18. Liao, J. *et al.* A vanadium-based metal-organic phosphate framework material $K_2[(VO)_2(HPO_4)_2(C_2O_4)]$ as a cathode for potassium-ion batteries. *Chem. Commun.* **55**, 659-662 (2019).

19. Wang, X. *et al.* Earth Abundant Fe/Mn-Based Layered Oxide Interconnected Nanowires for Advanced K-Ion Full Batteries. *Nano Lett.* **17**, 544-550 (2017).
20. Chong, S. *et al.* Cryptomelane-type MnO_2/carbon nanotube hybrids as bifunctional electrode material for high capacity potassium-ion full batteries. *Nano Energy* **54**, 106-115 (2018).
21. Tian, B., Tang, W., Su, C. & Li, Y. Reticular $V_2O_5{\cdot}0.6H_2O$ Xerogel as Cathode for Rechargeable Potassium Ion Batteries. *ACS Appl. Mater. Interfaces* **10**, 642-650 (2018).
22. Clites, M., Hart, J. L., Taheri, M. L. & Pomerantseva, E. Chemically Preintercalated Bilayered $K_xV_2O_5{\cdot}nH_2O$ Nanobelts as a High-Performing Cathode Material for K-Ion Batteries. *ACS Energy Lett.* **3**, 562-567 (2018).
23. Lin, B. *et al.* Birnessite Nanosheet Arrays with High K Content as a High-Capacity and Ultrastable Cathode for K-Ion Batteries. *Adv. Mater.* **31**, 1900060 (2019).
24. Chong, S. *et al.* Mn-based layered oxide microspheres assembled by ultrathin nanosheets as cathode material for potassium-ion batteries. *Electrochim. Acta* **293**, 299-306 (2019).
25. Zhang, H. *et al.* Enhanced K-ion kinetics in a layered cathode for potassium ion batteries. *Chem. Commun.* **55**, 7910-7913 (2019).
26. Hyoung, J., Heo, J. W., Chae, M. S. & Hong, S. T. Electrochemical Exchange Reaction Mechanism and the Role of Additive Water to Stabilize the Structure of $VOPO_4{\cdot}2H_2O$ as a Cathode Material for Potassium-Ion Batteries. *ChemSusChem* **12**, 1069-1075 (2019).
27. Gao, A. *et al.* K-Birnessite Electrode Obtained by Ion Exchange for Potassium-Ion Batteries: Insight into the Concerted Ionic Diffusion and K Storage Mechanism. *Adv. Energy Mater.* **9**, 1802739 (2019).
28. Zhao, S., Yan, K., Munroe, P., Sun, B. & Wang, G. Construction of Hierarchical $K_{1.39}Mn_3O_6$ Spheres via AlF_3 Coating for High-Performance Potassium-Ion Batteries. *Adv. Energy Mater.* **9**, 1803757 (2019).
29. Deng, L. *et al.* Layered Potassium Vanadate $K_{0.5}V_2O_5$ as a Cathode Material for Nonaqueous Potassium Ion Batteries. *Adv. Funct. Mater.* **28**, 1800670 (2018).
30. Jo, J. *et al.* Potassium vanadate as a new cathode material for potassium-ion batteries. *J. Power Sources* **432**, 24-29 (2019).
31. Zhu, Y.-H. *et al.* Reconstructed Orthorhombic V_2O_5 Polyhedra for Fast Ion Diffusion in K-Ion Batteries. *Chem* **5**, 168-179 (2019).
32. Vishnuprakash, P., Nithya, C. & Premalatha, M. Exploration of V_2O_5 nanorod@rGO heterostructure as potential cathode material for potassium-ion batteries. *Electrochim. Acta* **309**, 234-241 (2019).
33. Luo, Y. *et al.* Potassium titanium hexacyanoferrate as a cathode material for potassium-ion batteries. *J. Phys. Chem. Solids* **122**, 31-35 (2018).
34. Zhang, C. *et al.* Potassium Prussian Blue Nanoparticles: A Low-Cost Cathode Material for Potassium-Ion Batteries. *Adv. Funct. Mater.* **27**, 1604307 (2017).
35. Jiang, X., Zhang, T., Yang, L., Li, G. & Lee, J. Y. A Fe/Mn-Based Prussian Blue Analogue as a K-Rich Cathode Material for Potassium-Ion Batteries. *ChemElectroChem* **4**, 2237-2242 (2017).
36. Xue, L. *et al.* Low-Cost High-Energy Potassium Cathode. *J Am. Chem. Soc.* **139**, 2164-2167 (2017).
37. Bie, X., Kubota, K., Hosaka, T., Chihara, K. & Komaba, S. A novel K-ion battery: hexacyanoferrate(ii)/graphite cell. *J. Mater. Chem. A* **5**, 4325-4330 (2017).

38. Heo, J. W., Chae, M. S., Hyoung, J. & Hong, S. T. Rhombohedral Potassium-Zinc Hexacyanoferrate as a Cathode Material for Nonaqueous Potassium-Ion Batteries. *Inorg. Chem.* **58**, 3065-3072 (2019).
39. Liao, J. *et al.* A potassium-rich iron hexacyanoferrate/dipotassium terephthalate@carbon nanotube composite used for K-ion full-cells with an optimized electrolyte. *J. Mater. Chem. A* **5**, 19017-19024 (2017).
40. He, G. & Nazar, L. F. Crystallite Size Control of Prussian White Analogues for Nonaqueous Potassium-Ion Batteries. *ACS Energy Lett.* **2**, 1122-1127 (2017).
41. Piernas-Muñoz, M. J., Castillo-Martínez, E., Bondarchuk, O., Armand, M. & Rojo, T. Higher voltage plateau cubic Prussian White for Na-ion batteries. *J. Power Sources* **324**, 766-773 (2016).
42. Chong, S. *et al.* Potassium ferrous ferricyanide nanoparticles as a high capacity and ultralong life cathode material for nonaqueous potassium-ion batteries. *J. Mater. Chem. A* **5**, 22465-22471 (2017).
43. Wang, J. *et al.* A bi-functional device for self-powered electrochromic window and self-rechargeable transparent battery applications. *Nat. Commun.* **5**, 4921 (2014).
44. Eftekhari, A. Potassium secondary cell based on Prussian blue cathode. *J. Power Sources* **126**, 221-228 (2004).
45. Zhu, Y. H. *et al.* Transformation of Rusty Stainless-Steel Meshes into Stable, Low-Cost, and Binder-Free Cathodes for High-Performance Potassium-Ion Batteries. *Angew. Chem. Int. Ed.* **56**, 7881-7885 (2017).
46. Xue, Q. *et al.* Polypyrrole-Modified Prussian Blue Cathode Material for Potassium Ion Batteries via In Situ Polymerization Coating. *ACS Appl. Mater. Interfaces* **11**, 22339-22345 (2019).
47. Wu, X., Jian, Z., Li, Z. & Ji, X. Prussian white analogues as promising cathode for non-aqueous potassium-ion batteries. *Electrochem. Commun.* **77**, 54-57 (2017).
48. Padigi, P. *et al.* Prussian Green: A High Rate Capacity Cathode for Potassium Ion Batteries. *Electrochim. Acta* **166**, 32-39 (2015).
49. Pei, Y., Mu, C., Li, H., Li, F. & Chen J. Low-Cost $K_4Fe(CN)_6$ as aHigh-Voltage Cathode for Potassium-Ion Batteries. *ChemSusChem* **11**, 1285-1289 (2018).
50. Shadike, Z. *et al.* Long life and high-rate Berlin green $FeFe(CN)_6$ cathode material for a non-aqueous potassium-ion battery. *J. Mater. Chem. A* **5**, 6393-6398 (2017).
51. Chen, L. *et al.* Ortho-di-sodium salts of tetrahydroxyquinone as a novel electrode for lithium-ion and potassium-ion batteries. *Electrochim. Acta* **294**, 46-52 (2019).
52. Chen, L. & Zhao, Y. Exploration of *p*-$Na_2C_6H_2O_6$-based organic electrode materials for sodium-ion and potassium-ion batteries. *Mater. Lett.* **243**, 69-72 (2019).
53. Ma, J. *et al.* Endowing CuTCNQ with a new role: a high-capacity cathode for K-ion batteries. *Chem. Commun.* **54**, 5578-5581 (2018).
54. Chen, Y. *et al.* Organic electrode for non-aqueous potassium-ion batteries. *Nano Energy* **18**, 205-211 (2015).
55. Xing, Z. *et al.* A perylene anhydride crystal as a reversible electrode for K-ion batteries. *Energy Storage Mater.* **2**, 63-68 (2016).
56. Fan, L., Ma, R., Wang, J., Yang, H. & Lu, B. An Ultrafast and Highly Stable Potassium-Organic Battery. *Adv. Mater.* **30**, 1805486 (2018).

57. Zhao, J., Yang, J., Sun, P. & Xu, Y. Sodium sulfonate groups substituted anthraquinone as an organic cathode for potassium batteries. *Electrochem. Commun.* **86**, 34-37 (2018).
58. Li, B. *et al.* Electrolyte-Regulated Solid-Electrolyte Interphase Enables Long Cycle Life Performance in Organic Cathodes for Potassium-Ion Batteries. *Adv. Funct. Mater.* **29**, 1807137 (2018).
59. Jian, Z., Liang, Y., Rodríguez-Pérez, I. A., Yao, Y. & Ji, X. Poly(anthraquinonyl sulfide) cathode for potassium-ion batteries. *Electrochem. Commun.* **71**, 5-8 (2016).
60. Fan, L., Liu, Q., Xu, Z. & Lu, B. An Organic Cathode for Potassium Dual-Ion Full Battery. *ACS Energy Lett.* **2**, 1614-1620 (2017).
61. Li, C. *et al.* Poly(N-vinylcarbazole) as an advanced organic cathode for potassium-ion-based dual-ion battery. *Electrochim. Acta* **297**, 850-855 (2019).
62. Gao, H., Xue, L., Xin, S. & Goodenough, J. B. A High-Energy-Density Potassium Battery with a Polymer-Gel Electrolyte and a Polyaniline Cathode. *Angew. Chem. Int. Ed.* **57**, 5449-5453 (2018).
63. Ding, Y. *et al.* A Liquid-Metal-Enabled Versatile Organic Alkali-Ion Battery. *Adv. Mater.* **31**, 1806956 (2019).
64. Zhou, M. *et al.* Polydiaminoanthraquinones with tunable redox properties as high performance organic cathodes for K-ion batteries. *Chem. Commun.* **55**, 6054-6057 (2019).
65. Tian, B. *et al.* Carbonyl-based polyimide and polyquinoneimide for potassium-ion batteries. *J. Mater. Chem. A* **7**, 9997-10003 (2019).
66. Han, J. *et al.* Investigation of $K_3V_2(PO_4)_3$/C nanocomposites as high-potential cathode materials for potassium-ion batteries. *Chem. Commun.* **53**, 1805-1808 (2017).
67. Han, J. *et al.* Nanocubic $KTi_2(PO_4)_3$ electrodes for potassium-ion batteries. *Chem. Commun.* **52**, 11661-11664 (2016).
68. Chihara, K., Katogi, A., Kubota, K. & Komaba, S. $KVPO_4F$ and $KVOPO_4$ toward 4 volt-class potassium-ion batteries. *Chem. Commun.* **53**, 5208-5211 (2017).
69. Kim, H. *et al*. A New Strategy for High-Voltage Cathodes for K-Ion Batteries: Stoichiometric $KVPO_4F$. *Adv. Energy Mater.* **8**, 1801591 (2018).
70. Park, W. B. *et al.* KVP_2O_7 as a Robust High-Energy Cathode for Potassium-Ion Batteries: Pinpointed by a Full Screening of the Inorganic Registry under Specific Search Conditions. *Adv. Energy Mater.* **8**, 1703099 (2018).
71. Zhang, L. *et al.* Constructing the best symmetric full K-ion battery with the NASICON-type $K_3V_2(PO_4)_3$. *Nano Energy* **60**, 432-439 (2019).
72. Lin, X., Huang, J., Tan, H., Huang, J. & Zhang, B. $K_3V_2(PO_4)_2F_3$ as a robust cathode for potassium-ion batteries. *Energy Storage Mater.* **16**, 97-101 (2019).
73. Wang, S., Cui, B., Zhuang, Q., Shi, Y. & Zheng, H. Synthesis and Electrochemical Performance of Cobalt-Doped $KMnF_3$ as Cathode Materials for Potassium Ion Batteries. *J. Electrochem Soc.* **166**, A1819-A1826 (2019).
74. Lu, K. *et al.* Rechargeable potassium-ion batteries enabled by potassium-iodine conversion chemistry. *Energy Storage Mater.* **16**, 1-5 (2019).
